# What Others Say

**Published:** 1880-09

**Authors:** 


### EDITORIAL, ETC.

*What Others Say.*—The Medical Journals of the country notice from time to time the doings of the dentists, and make their comments. The *Medical Record* a weekly journal published in New York, writing of the various dental conventions and associations which have been at work lately, says of the Dental Association of the United States, that "it is expected to be the representative body of the American Dentists. It will hold annual meetings in Washington, where its permanent secretary will reside. The Association aims at high scientific work, and will arrange at once to secure governmental recognition to the dental profession, and help from it in studying ethnological peculiarities in their relation to dentistry. The Smithsonian Institute promises aid in this direction."

The passage of the resolution requiring credentials or a diploma as a perquisite to membership, says the *Record*, will, it is thought, give an impulse to dental education. It refers to the opposition encountered in forming the Association, the attitude of the American Dental Association, that it was believed to have had a too local character, etc.; and proceeds to say that "any genuinely scientific work done by the dentists will be of value to the medical profession. If this new association, therefore, secures, as it promises, the performance of such work, its creation will be a matter of congratulation for us."

But the writer goes on to express the opinion that the examination of the reports of the various meetings referred to, "disappoints the hope of finding *any great scientific work* performed." He thinks that the wranglings about the relative values of cohesive and non-cohesive gold, which vexed the souls of the American Dental Association, ought to have been settled long ago. He would hardly have said that if he had been a dentist.

Refering to a paper on anæsthesia, which, he says, "was defined to be a paralyzation of nervous tissue,' and to the discussions on the action of nitrous oxide, and whether it oxydized or prevented the oxydization of the blood, "*the fact was brought out very strongly that the Association did not know anything about the matter whatever.*" Rather hard, this, on the Association! Does the editor of the *Record* propose to show us the light? We will be glad to learn from him. He quotes Dr. Barrett, as saying "we dentists should be honest. We are not medical men, and are not acknowledged as such by physicians. There is no way by which a student can become a member of the medical profession except through a medical college."

Going on to the New York meetings the editor of the *Record* says he finds even less evidence of work than at Boston. It is worth while to say, with regard to these meetings, that they were, at least the D. U. Association of the United States, merely preliminary and formative. He justly criticises the "parliamentary infelicities," which are the bane of American meetings everywhere. The article concludes as follows:

"It will probably strike any one who reads the reports of the dental conventions to which we have been referring, that American dentists are sadly deficient, as yet, in scientific as well as in parliamentary knowledge. Their meetings have, however, shown a commendable appreciation of deficiencies and desire for progress. And considerable work was done which will eventually secure scientific and educational advancement in dentistry."

That the dentists "feel their deficiency" is a satisfaction certainly, and if the *Record* has aided in making them more conspicuous by his rather merciless criticisms, may help us. One cannot help feeling however, in reading the report of the American Neurological Association, which held its sixth annual meeting in New York in June last, an abstract of which is published in the same number of the *Record* from which the above is drawn, that *their doctors* differ quite as redically as ours, on subjects one would suppose they ought to be able to settle, or should have settled long ago; and this is true of the discussions in all medical societies, for even on apparently the simplest subjects there is dispute and doubt, darkness and lack of knowledge; though we are willing to concede more than the *Record*, in charging that we, the dentists, know *nothing*, for the M. D.'s in question know at least a little. We will publish in October No. an editorial from the *Medical Record* for June 17th of this year, in which the writer reviews the "position of the dentist," and seems more liberal than after seeing the reports of the Boston and New York meetings of August. Perhaps he expected too much of us. The remark he makes relative to the demand for educated dentists is worthy of thoughtful pondering, for he writes: "It is a fact, however, that the demand for highly educated dentists is not yet great enough to justify more than a small proportion of that profession in getting a medical diploma." It certainly has not been the observation of most of us that those who come from medical colleges to us, *brought with them acquirements, or to us honor.* H.

